# S36. DIFFERENTIAL ENCODING OF SENSITIZATION AND CROSS SENSITIZATION TO PSYCHOSTIMULANTS AND ANTIPSYCHOTICS IN NUCLEUS ACCUMBENS D1- AND D2- RECEPTOR EXPRESSING MEDIUM SPINY NEURONS

**DOI:** 10.1093/schbul/sby018.823

**Published:** 2018-04-01

**Authors:** Davide Amato, Jasper Heinsbroek, Peter W Kalivas

**Affiliations:** Medical University of South Carolina

## Abstract

**Background:**

Nearly half of all individuals diagnosed with schizophrenia abuse addictive substances such as cocaine. Currently, the neurobiological mechanisms in patients with schizophrenia that lead to cocaine abuse are unknown. A possible explanation for the co-morbidity between schizophrenia and addiction is that the rewarding properties of cocaine reverse the diminished motivational drive caused by chronic antipsychotic regimen. Moreover, chronic antipsychotic treatment can sensitize and amplify cocaine rewarding effects and exacerbate psychoses.

**Methods:**

The rewarding properties of cocaine are attributed to the differential effects of dopamine on D1 and D2 receptor-expressing medium spiny neurons (MSNs) in the nucleus accumbens (NAc). Using in vivo Ca2+ miniature microscopic imaging, we characterize the role of D1 and D2 MSN in mono- and a cross- sensitization paradigms. D1- and D2-Cre mice were injected with a Cre dependent calcium indicator (gCaMP6f) and implanted with a gradient index (GRIN) lens above the nucleus accumbens and calcium activity was recorded using a head mounted miniature microscope. Cocaine sensitization was measured after a classic repeated cocaine regiment and antipsychotic and psychostimulant cross-sensitization was measured by a single cocaine injection after chronic pre-treatment with haloperidol.

**Results:**

We found that both D1-MSN and D2-MSN populations are modulated by initial cocaine experience and further modulated during the expression of cocaine sensitization. A subpopulation of D1-MSN displayed initial activation, but reduced activity during the expression of sensitization. By contrast, the majority of D2-MSNs were suppressed by initial cocaine experience, but became active during the expression of sensitization. Furthermore, activity of D1- and D2-MSNs bidirectionally related with the observed behavioral responses to cocaine. Cross-sensitization following haloperidol treatment led to increased behavioral responses to psychostimulants. Current experiments are set out to investigate the neuronal responses of D1 and D2-MSN during cross sensitization between haloperidol and cocaine.

**Discussion:**

Cocaine sensitization leads to differential neuronal responses in D1- and D2-MSN and these responses are differentially correlated with the magnitude of the sensitized behavioral response. These results reveal important new insights in the neurobiological processes in the nucleus accumbens that underlie psychostimulant sensitization and provide an important new model for studying the pharmacology of antipsychotic effects on striatal function and its potential role in increasing the susceptibility of schizophrenic patients to developing drug addiction.

